# “You give everything the child requires to attend school”: Exploring caregiver involvement in children’s education in Ghana

**DOI:** 10.12688/openreseurope.20510.1

**Published:** 2025-09-16

**Authors:** Esinam Ami Avornyo, Richard Appiah, Elisabetta Aurino, Sharon Wolf

**Affiliations:** 1 Institute of Education, University of Cape Coast College of Education Studies, Cape Coast, Central, Ghana; 2 Department of Psychology, Northumbria University, Newcastle upon Tyne, England, UK; 3 College of Health Sciences, University of Ghana, Accra, Greater Accra Region, Ghana; 4 School of Economics, University of Barcelona, Barcelona, Spain; 5 Graduate School of Education, University of Pennsylvania, Philadelphia, Pennsylvania, USA

**Keywords:** caregiver involvement, educational engagement, engagement barriers, Ghana, learning

## Abstract

**Background:**

Parents’ and other primary caregivers’ involvement in children’s education is a major goal of education systems worldwide. However, the current literature lacks a broad understanding of what educational involvement means for parents in West African settings, and the factors that drive or limit their involvement.

**Methods:**

We examined caregivers’ views on their roles and involvement in and barriers to child education with 132 caregivers from rural and peri-urban communities in three regions of Ghana, using focus group discussions.

**Results:**

Most caregivers limited their involvement in providing basic and material resources. However, caregivers with a higher education discussed supporting their children’s homework. Four types of barriers to caregiver involvement were identified: social and economic, caregiver and family, educational system, and child-level factors.

**Conclusion:**

Our findings can inform context-tailored interventions and policies aimed at improving caregiver involvement, which is a key driver of educational outcomes.

## Plain language summary

In this study, we aimed to understand caregivers’ views on their roles and involvement in their children’s education, and the factors that limit their involvement. We conducted a focused-group discussion with 132 caregivers selected from rural and peri-urban communities across three regions in Ghana. Most caregivers felt that their role in their children’s education was to provide their basic needs and school materials. However, caregivers with higher educational levels supported their children’s homework. Furthermore, we identified four factors that limit caregivers’ involvement in their children’s education: social and financial issues; factors related to caregivers and their families; issues with the education system; and factors at the child level. These findings can help to develop interventions and policies to improve caregiver involvement in Ghana and other similar contexts.

## Introduction

Parents and other primary caregivers (hereafter referred to as caregivers) play a crucial role in fostering children’s development. In particular, their involvement in children’s education, including school- and home-based practices, is a major goal of educational systems worldwide ( Goodall, 2017; Smith, 2021). This is because caregivers’ educational involvement, such as attending parent-teacher meetings and home-based activities including cognitive stimulation, discussions about school progress and educational aspirations, and helping with homework, benefits children’s development, academic outcomes, and overall well-being ( Brossard *et al.*, 2020; Chowa *et al.*, 2013; González & Jackson, 2013; Nyarko, 2011).

In Ghana and other low- and middle-income countries (LMICs), caregivers value education as a major route out of poverty and have very high educational aspirations for their children ( Bernard *et al.*, 2019). However, their involvement in their children’s educational endeavors is generally limited, particularly for caregivers with lower educational attainment ( Chowa *et al.*, 2013). For instance, in a survey conducted in two regions of Ghana, only 13% of caregivers reported engaging in developmentally appropriate activities with their children in the previous three days ( Amadu *et al.*, 2018).

While barriers such as poverty, low caregiver education, and limited time can explain limited caregiver educational engagement, low levels of involvement in LMICs may be partially explained by how engagement is measured. Caregivers’ understanding of and involvement in educational activities are also partly culturally motivated in ways that may not be captured in current assessments ( Mundt *et al.*, 2015). Parents may engage in their children’s education and broader development in ways that are not fully captured by the scales commonly included in surveys that focus on cognitive and social-emotional stimulation.

The current literature lacks a broad understanding of what educational involvement means for parents in West African settings, and the factors that drive or limit their involvement. Existing evidence on barriers has highlighted the importance of resource and time constraints ( Mendez, 2010; Park & Holloway, 2013), parental education levels ( Aurino & Wolf, 2024; Skaliotis, 2010), and cognitive and behavioral barriers ( Hornby & Lafaele, 2011; Mayer *et al.*, 2020). Furthermore, parents may lack knowledge or have a limited understanding of the foci of educational systems, which in turn may limit their involvement ( Alieva, 2021). For instance, in recent years, educational reforms have occurred at different levels in Ghana, including the introduction of a new curriculum (i.e., a standards-based curriculum) that emphasizes active and child-centered teaching and learning approaches that differ from caregivers’ own experiences and expectations. Caregivers’ inability to understand these changes may hinder their involvement, which can be exacerbated for those with little or no education. Furthermore, parents may have limited guidance from teachers or experience stigma and discrimination by educators ( Balarin & Cueto, 2007).

The literature on the barriers to caregiver involvement in children’s education has mostly been explored by focusing on caregivers’ characteristics and the circumstances they experience (e.g., poverty, location). However, it is possible that factors related to their children, such as age, sex, and motivation, also shape involvement and interact with the caregivers’ characteristics. In an early review of the literature, Desforges and Abouchaar (2003) found that caregivers checking their child’s homework decreased as the child grew older. Similarly, Powell *et al.* (2012) found a decrease in caregiver-child cognitive stimulation during a child’s transition from preschool to kindergarten and kindergarten to first grade, but an increase in their provision of learning resources. In a recent synthesis of the literature on caregiver involvement in Europe, Alieva (2021) found that the frequency and intensity of parental involvement in school-based activities decreased when children transitioned from primary to secondary school levels. Importantly, the vast majority of this work has been conducted in high-income Western countries, leaving a limited picture of caregiver involvement across age groups in Africa or, more broadly, in LMIC contexts. In the latter, caregiver involvement may vary according to the child’s sex because of greater opportunity costs of schooling for girls (e.g., larger involvement of girls in household or care-work), lower perceived returns to girls’ education, and widespread gender bias in social norms and aspirations ( Adetunde & Akampae Akensin, 2008; Kainuwa *et al.*, 2013). They may also vary by a child’s age, as adolescents often take up work responsibilities alongside school.

Although these patterns offer a diverse picture of caregiver involvement, previous studies have been limited to quantitative research methods that evaluate the association between caregiver engagement and child outcomes. This limitation has provided few opportunities to understand the documented variations from the caregivers’ perspectives. Furthermore, little is known about caregivers’ views regarding their involvement and what caregiver engagement entails in majority world settings, such as Ghana, where caregiver education levels are often lower, learning environments can be of low quality, and many students are first-generation learners.

### The Ghanaian context

Over the last decade, the Ghanaian government has made efforts to improve access to basic education for all children, with the gross enrolment rate increasing from approximately 60% in 2010 to 103% in 2020 ( Ghana Education Sector Report, 2023). However, learning outcomes remain low. For example, according to the 2020 Ghana Education Facts, only 21% of learners, with almost equal proportions of males (20%) and females (22%), demonstrated the expected foundational reading skills. Similarly, only 16% of learners, with an almost equal share of males (17%) and females (14%), demonstrated the expected foundational numeracy skills. Notable regional disparities in learning gaps were observed, with Greater Accra having the highest proportion of children with foundational reading and numeracy skills, whereas the northern region had the lowest share. This emphasizes the need to understand caregivers’ perspectives across a diverse range of communities within Ghana.

Previous research has indicated that Ghanaian caregivers’ involvement in their children’s education and learning is low. Data from MICS 2017/2018 revealed that only 65% of children received support for their homework ( Ghana Statistical Service, 2018). Additionally, the same data showed that only 55% of caregivers met teachers at school to discuss their children’s progress. Although the Ghana government is taking steps to improve caregiver involvement through initiatives such as the Ghana Accountability for Learning Outcomes (GALOP) framework ( Ministry of Education, n.d.), for example, much less is known about how caregivers perceive their involvement.

## The present study

Relying on rich qualitative data, this study contributes to the global literature on caregiver engagement by examining Ghanaian caregivers’ perceived roles, contributions to their children’s educational endeavors, and barriers to their involvement. This knowledge is critical for future interventions aimed at increasing caregiver involvement in Ghana and potentially other West African and Sub-Saharan African settings. While research from Western contexts has identified multiple barriers to caregiver involvement (primarily focused on caregiver-level barriers), relatively little work has been published on the sub-Saharan African region, where cultural, social, and economic circumstances differ considerably. Furthermore, we examined engagement in relation to children’s characteristics, including age and sex, which is a novel contribution to the literature. Our results provide a framework for understanding the different barriers caregivers face and offer insights for designing context-tailored interventions and policies aimed at improving caregiver involvement.

### Research objectives

The present study draws on focus group discussions (FGDs) with 132 caregivers across six diverse communities in three regions of Ghana to explore caregivers’ perceptions of their role in their children’s education, as well as their perceived barriers to engaging in the way they preferred. A deeper understanding of how parents view their involvement is needed to successfully engage them in efforts to improve their children’s education. The goals of the FGDs were to examine (1) what constitutes educational engagement for parents and primary caregivers in Ghana; (2) to what extent engagement differs in relation to parents’ educational levels, as well as children’s sex and age; and (3) the primary perceived barriers to educational engagement.

## Methodology

### Ethical considerations

Our research was conducted in accordance with the Declaration of Helsinki on human subject research that emphasizes privacy, confidentiality, voluntary and informed consent, and the role of research ethics committees in reviewing and approving the research protocols. This study and its related protocols were reviewed and approved by the Ghana Health Service Ethics Review Committee (approval number: #GHS-ERC: 013/12/21). All participants were fully informed of the purpose of the study, potential risks and benefits, confidentiality, anonymity, and the voluntary nature of the study. The participants consented to participate in the study verbally and by signing or thumb-printing the consent form. The approval for the study spanned from January 17, 2022 to January 16, 2023. It is important to clarify that this study involved only caregivers of children aged 5–15 years and not their children.

### Sampling procedure

A sample of caregivers of school-aged children (age 5–15 years) was recruited from six purposefully selected rural and peri-urban communities. Preliminary census data from the 2021 Population and Housing Census indicate that 42.65% of Ghana’s population resides in rural settings with an average of 210 households. These households have scores below 50% of the poverty line (US$1.90/day) ( Ghana Statistical Service 2021a).

*Community selection*. Based on the three geographical blocs (northern, middle, and southern), we randomly selected two regions in each bloc to participate in the study using a list of rural and peri-urban communities obtained from the Regional Health Directorate. We randomly selected one rural community and one peri-urban community from the northern belt, one rural cocoa-growing community from the middle belt, and two rural communities and one peri-urban community from the southern belt. The sample was intentionally and evenly spread across Ghana to reflect cultural, agro-ecological, and socioeconomic variations across regions that may impact caregivers’ engagement in education. We focused on rural and peri-urban communities because they are characterized by the highest poverty rates and lack of infrastructure, including educational facilities.

*Participant selection*. We adhered to the community entry processes recommended for researchers conducting community-based participatory research in the rural context of Ghana ( Appiah, 2020; Appiah, 2021) to recruit participants from each community using a systematic sampling method, wherein every third and fifth household was selected in peri-urban and rural communities, respectively. A community elder familiar with the community terrain and a native speaker were recruited and trained as an independent mediator to lead the recruitment process, which involved seeking permission from the head of household and introducing the study to members of the household. Male and female members of the household aged between 18 and 60 years who were parents or primary caregivers of young children and adolescents aged five–15 years were eligible for inclusion. Primary caregivers included grandparents, relatives, and non-relatives responsible for the children’s development. We recruited 22 individuals from each community, comprising 11 males and 11 females, yielding a total of 132 participants.

### Sample characteristics

Table 1 presents the sample characteristics. The average age was 42.4 years. On average, caregivers had more than two children, and the majority of their children (96%) were currently enrolled in school, reflecting the nearly universal rate of primary school enrolment in Ghana. Caregivers were mostly children’s parents, but in some cases, they were either grandparents or other relatives such as older siblings. Finally, over half of the caregivers had either no education (34%) or a primary education (27%). The remaining participants attained junior secondary (29%) or higher levels of education.

**Table 1.** Sample characteristics.

	n	Mean or %	SD	Max	Min
**Caregivers characteristics**					
Male	126	48%	–	0	1
Age	125	42.36	10.65	21	70
Total no. children	126	2.7	1.21	1	7
No. of female children	126	1.2	1.03	0	4
No. of male children	126	1.4	0.89	0	4
Share of enrolled children	126	96.2	13.97	0	100
**Relationship to children**					
Mother	61	48%	–	0	1
Father	55	44%	–	0	1
Grandparent	7	6%	–	0	1
Other relative	3	2%	–	0	1
**Region**					
Brong Ahafo	22	17%	–	0	1
Central	18	14%	–	0	1
Eastern	22	17%	–	0	1
Greater Accra	19	15%	–	0	1
Northern	22	17%	–	0	1
Upper East	23	18%	–	0	1
**Level of education**					
None	43	34%	–	0	1
Primary	34	27%	–	0	1
Junior High School	36	29%	–	0	1
Senior High School	10	8%	–	0	1
Tertiary	3	2%	–	0	1

### Community characteristics

Except for one community in the southern belt, all the communities had an average of 150 households. Household incomes in these communities are below 50% of the poverty line (US$1.90 a day), classified as ultra-poor (US$ 1.25 or less a day; Ghana's District League Table, 2016; Ghana Statistical Service (GSS), Ghana Health Service (GHS), & Inner-City Fund (ICF) International, 2015). In all communities, residents spoke a local language, were primarily engaged in farming and trading, and were collectivistically socially oriented, governed by an elected chief and elders ( Appiah, 2020). Additionally, in all communities, road networks were poor, residents mostly used public toilet facilities and had access only to primary schools. While four communities had electricity, only approximately half of the households were connected to the grid, partially because of the associated costs.

### Focus group protocol

Focus group discussions (FGDs) were guided by our research objectives, focusing on the perceived roles of and barriers to educational engagement. Each focus group had an average of 10 participants. Participants discussed whether and how they engaged in their children’s education and the barriers they experienced with respect to engagement in their children’s education, including their views on girlchild education. Each FGD lasted approximately one and half hours, on an average.

### Data collection procedures

All field staff had a minimum bachelor’s degree, had previous experience conducting FGDs, and were native speakers of the main language spoken in the communities from which they were sampled. Prior to data collection, a two-day training session was organized for field staff, focusing on skill acquisition in community entry procedures, participant recruitment processes, facilitating FGDs, and research ethics issues. Trained independent mediators acted as community navigators to disseminate study information and protocols, supported the research team in seeking permission from gatekeepers/heads of households, and led the informed consent process and recruitment of individuals who agreed to participate. The independent mediator, working alongside trained research assistants, explained the purpose of the study, its benefits, and potential risks to all selected individuals during recruitment in their native language. Each prospective participant was given two days to seek further information and clarification from an independent mediator and a research team. If they wished, they could also discuss the invitation with family and friends as part of the decision-making process ( Appiah, 2021). Verbal and signed or thumb-printed informed consent was sought through an independent mediator and research assistant in the presence of a witness in the home environment of participants to ensure privacy, confidentiality, and safety of participants.

### Data analysis

All FGDs were digitally voice-recorded, and enumerators took extensive field notes, engaged in member checking, triangulation, and prolonged engagement with the data to ensure that the results were credible and reflected the participants’ perspectives ( Farrelly, 2013). The research team also conducted a data audit concerning data collection, analysis, and interpretation to maintain dependability ( Braun & Clarke, 2006). Research associates with bilingual (local language-English) competence and training translated and transcribed the interview data, which was conducted in the local language, into English using the dynamic equivalence approach ( Di & Nida, 2006). For specific terms and concepts, the translators consulted independent mediators from the specific community for clarification when necessary. The translated scripts were cross-checked with the audio data to ensure that the vocabularies and concepts in the translated text represented the participants’ expressions and corresponded with the context of the study.

The first author analyzed the data, adhering to the principles of deductive and inductive thematic analysis ( Braun & Clarke, 2006). Deductive coding was performed using pre-specified categories based on the research objectives, such as caregiver involvement and barriers. Inductive coding, inspired by grounded theory, was used to generate the individual codes. Related codes were then combined and grouped into sub-themes. The codes were subsequently reviewed to ensure a proper fit within each sub-theme. The remaining authors independently verified the assignment of codes and sub-themes.

## Findings

The results are presented under two themes: (a) caregivers’ perceived role and contributions to children’s education, and (b) barriers to caregiver involvement. In the first section, we also explore how caregivers’ roles vary according to their education, sex, and children’s sex and age.

### Perceived roles and contributions to children’s education

Three key sub-themes emerged that describe caregivers’ perceived role in their children’s education: i) *Tackling basic needs*; ii) *Offering general advice and encouragement to their children*; and iii) *Engagement in learning at home and school*.

***Tackling basic needs***. Most caregivers indicated that their key role in supporting their children’s education was to provide for their children’s basic needs. Essentially, they felt responsible for ensuring that children were well-fed, clean, enrolled in school, and had basic school supplies such as a tidy uniform, books, and stationery. The provision of such basic needs emerged as a central theme and was consistent among participants regardless of their level of education. For instance, one male caregiver noted, “ *Our role is to supply everything that the child needs for school*” (Male R10, Eastern). Other caregivers provided in-depth comments on their perceived role in addressing children’s basic needs, as evidenced by the following quotes:

My role as a caregiver is to bathe the children and get them breakfast before they leave school. I am to ensure they have pens, pencils, and exercise books at all times so that they can actively participate in class activities. (Female R2, Upper East).

Caregivers’ responses also demonstrated their understanding of the linkages between health, nutrition, and children’s learning, highlighting how a lack of these factors can undermine children’s capacities in school. One male caregiver mentioned, “ *The health of the child is the most important thing, another important thing is food because without health, the child cannot stay in school. When you are able to give your child a good feeding the child will do well in school* (Male R4, North). This was echoed by a female caregiver in the Upper East region, *“It is my duty as a parent to provide the child’s breakfast before sending him to school. If the child does not eat in the morning, he may not be able to concentrate in class, which will in the end negatively affect the child’s education* (Female R4, Upper East).

Caregivers also demonstrated knowledge of the importance of fulfilling these basic needs to ensure that a child is incentivized to attend school and enjoy their experiences in the classroom. The following narrations exemplify this awareness.

Often, children who wear ragged uniforms are hesitant to attend school, especially girls aged 13 and above. You give everything that the child requires to attend school, including uniforms, shoes, and books. This helps the child to feel at ease in school. (Male R6, Accra).
You need to provide the child with a school uniform even when you do not have the means to do so. Some children go to school with patched uniforms, and they may not be happy wearing patched uniforms. (Male R11, Eastern).

***Offering advice and encouragement***. The caregivers offered advice and encouragement to their children as a strategy to motivate their children’s education. While this role did not differ based on the caregiver’s level of education, male caregivers frequently mentioned it as a key role compared with female caregivers. The advice spanned reminding children about the importance of regular school attendance to listening to their teachers and emphasizing the significance of education as a way out of poverty. This is evidenced by the accounts of two male caregivers.

[…] While the child is young, say five years old and above, keep encouraging the child to aim high, indicating your dissatisfaction over your own life and how you want the child to do better than you have done … When the child fails at school you do not have to insult the child but rather encourage and advise the child to do better next time. If you keep doing that the child strives to do better next time (Male R8, Eastern).
All of us here have not had education, so we have been advising our children to be serious and become better than us in the future because farmland is no longer available for farming. (Male R8, North).

Caregivers also offered advice to the older children. Female children were advised to remain in chaste to prevent early pregnancy, while male children were often advised to abstain from health risk behaviors, such as drinking and smoking. For instance, one male caregiver stated,

For a female who is approaching puberty or has already entered that phase of her life, you educate her on the dangers of getting involved with the opposite sex, which can result in an abrupt end to her education should she get pregnant …With the boy, you advise him not to smoke. years (Male R4, Eastern).

This was also echoed by a female caregiver: *“With the girls I advise them on how to be responsible so that they don’t get pregnant as teenagers. I also advise boys to desist from companies that can lead them to drink and smoke*” (Female R10, Middle).

***Engagement in learning at home and school***. Caregivers recounted engaging and supporting their children’s education both at home and at school. At home, caregivers reported supporting their children with their homework. However, this was frequently mentioned by caregivers with formal education, and a few with no formal education. Nonetheless, caregivers with no formal education and those with primary and junior secondary education supported learning by reminding their children to complete their homework or seeking help from family (e.g., other siblings), neighbors (if they had some educational attainment), or the child’s classmates: *“It is also my duty to ask the child when he returns from school whether he was given homework. And if there is homework for him, I will remind him to complete it after eating”* (Female R4, Upper East).

I also have to ask the children what they learned when they return from school. If they struggle to remember what they have learned, I have to get their classmates who are good to help my children revise what they have learned in school. (Male R9, Upper East).
Our role as a parent in supporting children’s education is to inspect their homework. If you cannot help, ask someone to help. (Female R8, Accra).

In contrast, caregivers with higher educational attainment (senior secondary and above) indicated providing direct assistance with homework, as one female caregiver stated, “After school, it is my duty to ask them what they learned in school for the day and assist them in completing their homework, if any” (Female R2, Upper East).

Concerning school-based activities, the majority of caregivers recounted visiting their children’s schools to check their children’s attendance/truancy and to discuss progress with their teachers. Of note, only caregivers with formal education indicated that they had visited their children’s schools for these engagements. For those who did, it was an important strategy to stay abreast of their child’s progress and to understand how they can adequately support their children’s academic success. This is exemplified by the following quote.

In addition, we have to visit the children at their schools to determine whether they are in school. If they are at school, we have to find out how they are doing academically. This will help parents appropriately advise their children. Some of the children may not go to school, but the visit will help us to identify these children so that we can deal with them appropriately. (Male R4, Upper East).
If your child is in school, you have to visit to know about their studies…Once in a while, you have to try your best to go to the school to see if your children really go to school, whether they study, and how their studies are (Female R1, Accra).

### Is perceived caregiver role equal by child’s sex and age?

The findings suggest that caregivers believe that they provide equal support to all children irrespective of their sex or age. One male caregiver narrated: “These children, they are all given to you by God, whether they are boys or girls, young or old, they are all equal and they deserve equal support. So, my support for these children is not based on their sex or on their age. You can’t separate their needs” (Male R2, North).

However, some caregivers acknowledged that the girls did not receive the same level of support historically. While this is no longer perceived as the case, past pro-boy bias was ascribed to sociocultural and economic factors:

They are all children, and the sex of a child does not make him or her more special than the other. However, culturally, we understand that our daughters are not permanent members of our families since they will move to stay with their husbands. However, if they excel academically, it will also be beneficial. (Male R5, Upper East).
[…] Our fathers used to say that they would rather educate their sons instead of their daughters because the girls can end up with pregnancy at any given time so they would not waste their money in on a girl’s education. Today there is no such thing. We don’t even hear of such cases. (Female R7, Eastern)

Although caregivers generally agreed about the need to provide support for both younger (5–9 years) and older (10–15 years) children alike, irrespective of sex, they nonetheless acknowledged that the level of support required by the age group and child’s sex may differ. There were differences in the types of support provided to children of different ages. Most caregivers mentioned that younger children generally required more attention, care, support, and encouragement than older ones, as the latter *“should already know what is right or wrong”* (Male R7, Upper East). In particular, the majority of caregivers indicated that providing care and encouragement for younger children is essential to instilling their interest in school.

Younger children need more attention. You must see to it that they develop a positive attitude towards school; else, when they get a bit older because you did not teach them to be positive about their education, they could go wayward when they got older (Male R2, Central).

Caregivers felt that older children required more economic support than younger children, as educational costs increased with age. The following quote illustrates participants’ views:

Please, there is a little difference, because if the child is between 5 and 9 years old and about to go to school, you would put fruit juice, biscuit, and other things. When the child is 10 years old, you will not buy those things, but the school fee is higher than when the child is 5 to 9 years old (Female R6, Accra).

Most caregivers agreed that, in the case of adolescent girls, educational and other costs were even higher than for adolescent boys, especially after puberty.

For me, I will say that roles sometimes change. If the child is a girl, after 10 or 11 years, they start menstruating, and you have to buy pads and many panties for them in addition to their school needs. Boys do not use any of these devices. (Female R4, North).

In addition to the cost of menstrual care, adolescent girls are seen as particularly vulnerable to teenage pregnancy, thus requiring increased monitoring to safeguard their education and future, as this quote explains: “ *Yes, there are differences. Your girl child is approaching puberty and the physical indications that come with it around the age of ten. If you do not pay more attention to her. Men start to show interest in her. Her future can be cut short”* (Male R1, Accra). Increased caregiver monitoring emerged as a theme for adolescents generally, as older children were often seen as more likely to get into trouble and abandon school as compared with younger ones: “ *The difference is, you must keep an eye on the studies more than when the child was in primary school. In primary school, it is full of play, at the junior high secondary level you must keep an eye on the child”* (Female R3, Accra).

### Barriers to caregiver educational involvement

Barriers emerged around four themes: social and economic, caregiver and family, educational system, and child-level factors.

***Social and economic factors***. Financial hardship has emerged as a major barrier to caregivers’ engagement in education. In-depth accounts across all sites illustrated the daily challenges caregivers faced in ensuring that the family’s basic needs were met. Socioeconomic challenges remained large and pressing for caregivers, given that they identified the provision of basic needs (e.g., feeding, buying books, uniforms, stationarity, and paying school fees) as their main role in supporting child education.

Participants’ accounts also highlighted multiple challenges related to the seasonality of rural incomes, unpredictability of harvests based on the weather, and the low pay and precariousness of informal jobs in urban sites. These challenges hindered their ability to provide food for their children, as noted in the following quote:

Some parents could not afford breakfast for their children. When children are hungry, they are unable to concentrate on their learning. I called a child in this community a few days ago asking him why he was not in school. His reply was, I am very hungry and there’s no breakfast at home, and when I go to school on an empty stomach, I am not able to learn (Female R4, Upper East).

To meet their financial needs, some caregivers engage in long workdays or job searches. In their narrations, they reported engaging in informal jobs such as street vending and working on their farms from dawn to dusk, often without speaking to their children, because they would still be sleeping when they leave home for work. When they return home, they are often too tired to inquire about their children’s day at school or check on their homework, “ *Work and tiredness also make it difficult for us to help them do their homework”* (Male R8, Middle). Caregivers face difficulty in choosing between spending time working to provide for their families and spending time with their children. For all subgroups across the six communities, caregivers considered time with their child as a luxury. This is evidenced by the account of a male caregiver in the central region.

I am a driver, and I am not home most of the day. I wake up early, leave for work, and return late in the evening ... I do not even know when they retire to their bed at night. I return from work late and get tired. This has greatly affected their performance in school. A great part of that error can be attributed to me because I am unable to make time for them … I do not have the luxury of time. I am unable to attend P.T.A meetings. (Male R5, Central).

Another important factor hampering caregivers' engagement and children’s ability to study in the evening is the lack of electricity in their homes.

If there were electricity and the children brought homework from school, we would be able to plead with someone to assist the children in their homework. However, because there is no electricity, it is discouraging to call someone to assist them in the dark (Female R7, Middle).

This was echoed by a male respondent in the Middle belt, “ *If we can make progress, we need electricity. At least, if there was electricity, I could always help my children do their homework after returning from the farm in the evening”* (Male R6, Middle).

***Caregiver and family factors***. Caregivers with no or low education levels acknowledged the impact of their educational status on their ability to engage or support their children’s education. Many quotes, including the narration below, highlight a sense of frustration among caregivers regarding their inability to support their children with their homework, mainly because they could not read or write.

It hurts me that I am not educated. I cannot relate well to my children. If I had been educated, I would have had enough opportunities to help educate my children much better than I am doing now. If my children come back from school with any document or assignment, I have only two options: either ask the child to read and translate to my understanding or get a neighbor to read and translate for me (Female R3, Upper East).

The inability of caregivers to effectively engage in their children’s educational activities, including homework, due to their low educational background is perceived as a major factor contributing to lower learning outcomes among children. This could potentially lead to an intergenerational cycle of low education, as summarized in this quote:

Our educational level hinders efforts to engage in the education of children. Sometimes, we wish that we could assist the children in completing their homework assignments, but unfortunately, we are limited because some of us were never enrolled in school. The children end up doing what they think is the best. In the end, they obtain very poor grades, which demoralize and discourage them from continuing education (Female R2, Upper East).

Closely related to level of education is the lack of knowledge about the benefits of education by some caregivers. The majority of caregivers valued education, as their narrations often emphasized its significance and the high hopes they attributed to education as a fundamental route out of poverty. However, in a few cases, respondents acknowledged that their low level of education served as a barrier to engagement. For instance, a male respondent from the Eastern region mentioned that some caregivers, especially those with little or no formal education, did not fully appreciate the benefits of formal education. Nonetheless, such a limited awareness of the importance of education appears to be abating. For instance, one caregiver mentioned,

Some parents do not know the benefits of education. Most parents did not have any formal education; hence, they did not consider it important. They do not have any aspirations to take the child to school, and they are clueless about the aspiration of the child. For this reason, they struggle to take an interest in their child's education, even if they have money. (Male R5, Eastern).

***Educational system factors***. Caregivers with some level of education felt that the current homework assignments given to children were well above their educational level, which eventually discouraged them from actively showing interest in their children’s educational matters. Additionally, they felt that their current instructional methods differed from the pedagogy they had received. Caregivers complained about this change in the educational curriculum, which in turn prevented them from engaging and contributing to their children’s educational activities. This issue is highlighted in the following quote:

The current educational system hinders participation in children’s education. When we were in school and when you were given homework, the teacher solved the first question, which became an example for your parents to look upon to help you solve what is left. However, when children are given homework, there are no examples. How do I help my child complete the work? There has been a change in the educational system compared to what we have experienced (Male R4, Eastern).
It is true that parents are unable to support their children in their education. The things being taught today are difficult; I cannot even mention them…I do not know, so I do not get involved with it much. (Female R1, Accra).

Another significant barrier relates to the gradual shift from traditional in-person teacher-directed teaching and learning approaches to reliance on technological tools. Most caregivers have limited access to and knowledge of the use of devices, which in turn limits the level of support they offer to children’s education-related activities. The following quote also highlights the cost implications in terms of purchasing data to download instructional content from the Internet, which may potentially increase educational inequality.

[...] Now, if you don’t have a smart phone the child cannot do the homework. They say everything is online, as I sit here I don’t have a smart phone. Let’s say I can’t write and read, if my child brings homework home, how can I teach the child. It is a worry to us. (Female, R10, Accra).

***Child-related factors***. Another important barrier to caregivers’ involvement in their wards' education is low motivation or interest in school. This could partly be ascribed to previous low achievements and a lack of sufficient educational support.

If a girl’s academic performance is low and you cannot get her part-time teacher to help her, she might not be interested in education because of poor academic performance (Female R11, Middle).
Poverty has affected the education of our children in many ways, such that our children are unable to concentrate in class when teachers are teaching because of hunger. They also sometimes see themselves as inferior to children from better homes. (Male R5, Upper East).

To some extent, caregivers attributed the low motivation of their children to the lack of employment opportunities, even after the completion of tertiary education. Caregivers opined that some children become demotivated because other school leavers have failed to secure a job in the formal sector, thus serving as a precedent that children reference to justify their lackadaisical approach to school, as commented by several caregivers in the northern region.

These children always look at their senior brothers, who have completed school and have no jobs. So, when you always try to force them to go to school, they tell you to look at their brothers, who have completed school and are not working. Therefore, the government should create new job avenues. This can also motivate parents to engage well with their children and with school issues when they know that their children will become somebody in the future. (Male R1, North).

## Discussion

This study examined caregivers’ perspectives on their role and involvement in the education of school-age children (5–15 years) and barriers to their involvement in rural and peri-urban communities in Ghana. Generally, caregivers acknowledged the critical role they play in their children’s education, although some viewed their roles as limited to providing basic and material resources for their children. This is consistent with recent findings from another qualitative study in Ghana that explored parent and teacher perceptions of early childhood education and parent-teacher relationships ( Wolf, 2020).

### Caregiver roles and contributions to children’s education

Our findings show that caregivers, to a greater extent, were aware of the educational needs of their children and often made efforts to provide for both boys and girls and for children of all ages. Although caregivers across cultures provide for their children’s basic needs, the emphasis caregivers in the present study placed on meeting children’s food, health, and school costs is noteworthy. In other words, caregivers identified the provision of material resources as playing a key role in supporting children’s education. It is possible that this role may have been passed on from one generation to the next, and therefore remains critical in caregivers’ understanding of their involvement. However, caregivers were especially concerned about food and health, as evidenced in the following quote: “ *If the child does not eat in the morning, he may not be able to concentrate in class, which will in the end negatively affect the child’s education.”* This finding must be contextualized to the realities of millions of families in sub-Saharan Africa who struggle with food insecurity, poverty, and childhood malnutrition ( Black *et al.*, 2017). Caregivers were painstakingly aware of the adverse consequences of food insecurity on child learning, consistent with evidence that has associated hunger and malnutrition with hyperactivity, inattention, lack of energy to concentrate in class, and lower skills ( Aurino *et al.*, 2020; Ke & Ford-Jones, 2015; Wolf & Avornyo, 2023).

There were important differences in perceived involvement based on the caregiver education level. Caregivers with higher educational attainment discussed more direct engagement with their children’s education, either by actively participating in or reviewing their children’s homework as an additional part of their role in supporting their children’s education. By contrast, those with little or no education tended to only remind their children to do their homework. This finding is consistent with studies conducted in other LMICs (e.g., Ethiopia and India) and highlights the perceived lower self-efficacy of less-educated caregivers in supporting their children’s learning ( Portela & Atherton, 2020). Although caregiver education has long been a key predictor of children’s learning outcomes, the narration of caregivers in our study suggests why it matters in this context. We suggest that education and policy interventions should include school-based programs, such as increased in-school support, to allow schools to compensate for home disadvantages. Further, previous evidence highlights that parents often receive little guidance from schools on their children’s progress and how to support their children’s learning experiences ( Balarin & Cueto, 2007). Thus, providing more explicit guidance to caregivers on their children’s progress and how to support their children’s learning experiences would be highly beneficial, especially for caregivers with lower educational levels.

An important finding of this study is the observed sex differences in caregiver educational engagement: male caregivers were more likely than female caregivers to offer advice and encouragement to support their children’s education. While this finding is uncommon in the literature on caregiver engagement, it offers unique insights into how social and cultural practices influence caregiver involvement. In the relatively collectivistically socially oriented communities of Ghana, offering advice is considered a strong parenting tool and a cultural obligation of adults to children and younger folks, helping shape their development and instilling sociocultural values and norms ( Gyekye, 2010). Therefore, it is unsurprising that the participants considered their offer of advice to contribute to children’s education in this relatively patriarchal society. A study exploring parenting practices and stakeholders’ understanding of raising children in Ghana found that offering advice emerged as an important mode of enforcing positive values among children but did not explore differences by caregiver sex ( Essuman & Amo-Adjei, 2019). This insight could serve as valuable information to guide the targeting and design of context-specific school-based programs targeting caregiver school engagement, supporting caregivers (and males in particular) more explicitly in the type of advice and guidance they can provide to their children, even if they cannot directly support them in completing homework.

Another important finding of this study was that most caregivers acknowledged the need to support all children’s education equally, regardless of their age or sex. There were, however, divergent views about the type of support children of different ages and sexes needed. On one hand, caregivers recognized the importance of early caregiver support in terms of time and encouragement for younger children, particularly as a foundation for future academic success. On the other hand, caregivers felt that risk factors increased in adolescence and, therefore, additional guidance, monitoring, and investments are required at this stage. Caregivers generally found it necessary to engage in educational activities of both boys and girls. However, many of them highlighted that adolescent girls may face higher barriers to their education, due to perceived higher costs for supporting their needs compared with adolescent boys, higher opportunity costs of schooling given their higher involvement in house chores, and the risk of early pregnancy and subsequent school dropout. The notion of a generational shift towards equality of educational opportunities for boys and girls was also discussed, with caregivers highlighting the variations in how they perceived girls’ education compared to the previous generation of caregivers. However, the findings of the present study showed that both male and female caregivers were generally in support of girls’ education, contrary to earlier findings ( Ntim, 2013), despite the fact that sex-based disparities continue to exist in education, particularly in rural areas ( Liu, 2022). We surmise that this perspective may have been partly influenced by women’s empowerment in political and educational settings in recent times in Ghana. Presently, three Vice Chancellors in three leading public universities (including a few Pro Vice Chancellors), the vice-president of the ruling party, and a growing number of members of parliament are women. Most of these women are active in the media and may positively influence caregivers and adolescents. Understanding why gender gaps in education persist despite changing attitudes is an important area of future research.

### Understanding barriers to caregiver engagement

Another contribution of this study is that the results provide a framework for understanding barriers to caregivers’ educational engagement. The reported barriers fell into four categories: social and economic, caregiver and family, educational system, and child level.

Caregivers highlighted poverty and economic hardship as the main barriers to involvement in their children’s education, and the efforts of caregivers were clearly impacted by their socioeconomic limitations. For example, some caregivers recounted their dilemma of having to decide between investing their very limited savings in family farms to overcome poverty and food insecurity or buying school supplies for their children. In addition to monetary constraints, caregivers highlighted that the long hours they spent on their farms or in precarious urban jobs also limited their engagement with their children. This finding indicates the key role of social protection and job creation policies as pathways for improving educational involvement.

The caregivers in the present study also referred to their inability to catch up with changing educational systems, including new pedagogical practices and the use of technology, as barriers to their involvement. This finding is consistent with previous findings in Europe ( Alieva, 2021). Importantly, this issue becomes more challenging for caregivers with low or no education, given that their level of education already limits their involvement and may be connected with additional barriers (e.g., stigma/discrimination from teachers and more limited access to technology). While educational changes and reforms are inevitable, information about such changes and requirements can be communicated directly to caregivers. In particular, teachers can play an important role in reaching caregivers.

Finally, another barrier identified was children’s low educational motivation, as perceived by caregivers. On one hand, we found that low motivation arose from poor school performance. It is possible that caregivers and children lack useful information to keep them on track, as found in a study conducted in Peru ( Balarin & Cueto, 2007). Alternatively, the quality of teaching may be insufficient to promote learning and ultimately demotivate the children. This finding could motivate school-based interventions that support schools in tracking student performance regularly and directly target the performance and learning outcomes of children who are off-track (e.g., teaching at the right level). Schools can also initiate systematized programs to reach out to caregivers with explicit information on how they can support their children to get on track. On the other hand, the perceived (and real) difficulty associated with future employment opportunities is also seen as a factor contributing to reduced children’s education motivation and, hence, caregiver involvement. This concern is largely reflected in the country’s high unemployment rate ( Ghana Statistical Service, 2021b). Therefore, it is necessary for the government and policymakers to take crucial steps to address this to re-engender trust and motivation in the benefits associated with education.

## Limitations and conclusions

The findings should be interpreted with caution considering the limitations of this study. First, although we aimed to sample caregivers from a diverse set of regions and communities across Ghana, our sample was limited in terms of size and scope. Thus, our findings are not nationally representative and cannot be extrapolated beyond the included communities. Second, while the focus group discussions lend rich data that provide insights from caregivers themselves, we were limited to one source of data to draw conclusions. Multi-modal and multi-informant data, such as child reports on their experiences with their caregivers’ engagement (including barriers) and/or national survey data on caregiver perceptions and involvement, would help situate our findings in broader family and national contexts.

Nonetheless, our findings add important insights into the literature on education in LMICs and caregiver involvement, given their priority in school systems worldwide. Consistent with other findings from Ghana (e.g., Appiah, 2020), caregivers in our sample highly valued education and did what they felt they could to support their children’s success in school. However, our results suggest that efforts to increase engagement will have limited success without addressing the real barriers that caregivers face, including time constraints, economic hardship, limited knowledge of how to support children, and poor learning, which can demotivate children and caregivers. Ultimately, caregiver engagement cannot be considered in isolation and should be considered a key component, in addition to improvements in education and social welfare systems.

## Data Availability

The data underlying this study are not publicly available due to ethical and confidentiality concerns. Participants shared in-depth personal information about their lives and experiences, and the sensitive nature of this information – particularly involving vulnerable populations living in contexts of poverty – poses a risk of misuse if accessed beyond the intended scope of the research. The consent agreement that has been approved by the Ghana Health Services and administered to participants explicitly states that only core members of the research team may access the data. Public data sharing via repositories was not included in the study information sheet or consent process. To request access, please contact the corresponding author ( esinam.avornyo@ucc.edu.gh). Access may be granted after users provide a written justification and data use plan, sign a data-sharing agreement, and confirm that the data will be used solely for scientific research purposes, with no conflicts of interest among involved researchers. Zenodo. Exploring caregiver involvement in children's education in Ghana - Study materials. https://doi.org/10.5281/zenodo.15688949. ( Avornyo *et al.*, 2025) This project contains the following extended files: participant information sheet consent form interview guide Data files are available under the terms of the CC BY license.
