# Supplementary material for: “You give everything the child requires to attend school”: Exploring caregiver involvement in children’s education in Ghana
Source: Open Res Eur. 2026 Mar 12;5:283. Originally published 2025 Sep 16. [Version 2] doi: 10.12688/openreseurope.20510.2 (PMC12989055; doi:10.12688/openreseurope.20510.2)
Supplement: Peer review history

# Reviewer report: Reviewer response for version 2

**DOI:** 10.21956/openreseurope.25216.r71067

**Published:** 2026-03-14

**Authors:** Janet Goodall

**Affiliations:** 1 Swansea University Library, Swansea, Wales, UK

No further comments

If applicable, is the statistical analysis and its interpretation appropriate?

Not applicable

Is the study design appropriate and is the work technically sound?

Yes

Is the work clearly and accurately presented and does it engage with the current literature?

Partly

Are the conclusions drawn adequately supported by the results?

Partly

Are sufficient details of methods and analysis provided to allow replication by others?

Yes

Are all the source data and materials underlying the results available?

No source data required

Reviewer Expertise:

Parent, family and community engagement in learning

I confirm that I have read this submission and believe that I have an appropriate level of expertise to confirm that it is of an acceptable scientific standard.

# Reviewer report: Reviewer response for version 2

**DOI:** 10.21956/openreseurope.25216.r71066

**Published:** 2026-03-14

**Authors:** Charlotte Haines

**Affiliations:** 1 York St John University, York, England, UK

This is an excellent article. I am very grateful to the authors for being so open to my comments and amending the article accordingly. I think they have strengthened the precision and robust quality of their argument. I think it is also very readable to a variety of readers now. 

 I am particularly grateful to them making it so easy to see how they have responded. 

 Congratulations on such an important piece of research.

If applicable, is the statistical analysis and its interpretation appropriate?

Not applicable

Is the study design appropriate and is the work technically sound?

Yes

Is the work clearly and accurately presented and does it engage with the current literature?

Yes

Are the conclusions drawn adequately supported by the results?

Yes

Are sufficient details of methods and analysis provided to allow replication by others?

Yes

Are all the source data and materials underlying the results available?

No

Reviewer Expertise:

My PhD is about parent engagement, and I have published in this area.

I confirm that I have read this submission and believe that I have an appropriate level of expertise to confirm that it is of an acceptable scientific standard.

# Reviewer report: Reviewer response for version 1

**DOI:** 10.21956/openreseurope.22194.r60756

**Published:** 2025-10-10

**Authors:** Charlotte Haines

**Affiliations:** 1 York St John University, York, England, UK

This is a very interesting and important article and I am grateful to the author(s) for carrying out this research and reporting it so carefully. 

 The amendments I suggest will help strengthen it's criticality and authority but in effect are only minor. Most comments relate to the literature engagement and conclusions as the methodology is very strong and clear as are the findings which are reported exceptionally well and give a really voice to the participants. 

 I think it would help if in places there was a little more criticality in terms of thinking about the systemic issues present impacting the parents. There is quite a bit of western literature that demonstrates that it is systemic issues such as the amount of money. income that is available to parents this impacts the educational outcomes for children. See Gewirtz, 2001; Reay, 2006; 2008; Vincent, 2001; Vincent and Martin, 2002 for starters. 

 Please see the specific suggestions below:

 1. “The current literature lacks a broad understanding of what educational involvement means for parents in West African settings, and the factors that drive or limit their involvement. Existing evidence on barriers has highlighted the importance of resource and time constraints ( Mendez, 2010; Park & Holloway, 2013), parental education levels( Aurino & Wolf, 2024; Skaliotis, 2010), and cognitive and behavioral barriers…”               What are the barriers that are mentioned? What is meant by cognitive barriers – as this could be understood that the parents are not intelligent enough which I don’t think is true considering what else is said in the article.

 2. “Furthermore, parents may lack knowledge or have a limited understanding of the foci of educational systems, which in turn may limit their involvement ( Alieva, 2021).” It may be useful to engage with the work of Moll et al regarding funds of knowledge here. Also maybe reword as … lack knowledge of, or have limited... so it doesn’t look like you are saying lacking knowledge generally.

 3. There is a very good synthesis of literature but try and be geographically specific. Where are the studies you have mentioned located? Are they still relevant to Ghana. ( I am sure they are but the specificity will help credence and authority.)

 4 “Although these patterns offer a diverse picture of caregiver involvement, previous studies have been limited to quantitative research methods that evaluate the association be”

 what patterns?

 5. “gross enrolment rate increasing from approximately 60% in 2010 to 103% in 2020”

 I think I know what the author means but worth clarifying how it is 103%

 6. “However, learning outcomes remain low. For example, according to the 2020 Ghana Education Facts, only 21% of learners, with almost equal proportions of males (20%) and females (22%), demonstrated the expected foundational reading skills. Similarly, only 16% of learners, with an almost equal share of males (17%) and females (14%), demonstrated the expected foundational numeracy skills.  –

 what age are these learning outcomes applied to?

 7. “although caregiver education has long been a key predictor of children’s learning outcomes, the narration of caregivers in our study suggests why it matters in this context.”

 Although the biggest impact factor is arguably income rather than education. Systemic factors are key.

If applicable, is the statistical analysis and its interpretation appropriate?

Not applicable

Is the study design appropriate and is the work technically sound?

Yes

Is the work clearly and accurately presented and does it engage with the current literature?

Yes

Are the conclusions drawn adequately supported by the results?

Yes

Are sufficient details of methods and analysis provided to allow replication by others?

Yes

Are all the source data and materials underlying the results available?

No

Reviewer Expertise:

My PhD is about parent engagement and I have published in this area.

I confirm that I have read this submission and believe that I have an appropriate level of expertise to confirm that it is of an acceptable scientific standard, however I have significant reservations, as outlined above.

# Response

**Published:** 2026-02-25

**Authors:** Esinam Avornyo

**Affiliations:** Institute of Education, University of Cape Coast College of Education Studies, Cape Coast, Central, Ghana

Please see the specific suggestions below:

1. “The current literature lacks a broad understanding of what educational involvement means for parents in West African settings, and the factors that drive or limit their involvement. Existing evidence on barriers has highlighted the importance of resource and time constraints (Mendez, 2010; Park & Holloway, 2013), parental education levels (Aurino & Wolf, 2024; Skaliotis, 2010), and cognitive and behavioral barriers…” What are the barriers that are mentioned? What is meant by cognitive barriers – as this could be understood that the parents are not intelligent enough which I don’t think is true considering what else is said in the article.

**Response**: Thanks, we agree that systemic barriers are also key, so we have added references to these in our revised text. For the cognitive barriers, we did not mean at all lack of intelligence. This terminology is taken by the behavioral economics and behavioral science literatures, referring to limited bandwidth, present bias, etc., as documented by Mayer et al. (2019), among others. Here is our revised answer: *The current literature lacks a broad understanding of what educational involvement means for parents in West African settings, and the factors that drive or limit their involvement. Existing evidence on barriers has highlighted the importance of systemic barriers* *such as inadequate infrastructure (Kambona & Ndibalema, 2025), but also of parental resource and time constraints (Mendez, 2010; Park & Holloway, 2013), parental education levels* *- which is our proxy for socioeconomic status (Aurino & Wolf, 2024; Skaliotis, 2010), and cognitive and behavioral barriers such as limited bandwidth or present bias (Mayer et al 2019)*

1. “Furthermore, parents may lack knowledge or have a limited understanding of the foci of educational systems, which in turn may limit their involvement (Alieva, 2021).” It may be useful to engage with the work of Moll et al regarding funds of knowledge here. Also maybe reword as … lack knowledge of, or have limited... so it doesn’t look like you are saying lacking knowledge generally.

**Response**: Thanks for your insights. We have reworded this to reflect your suggestion: “Furthermore, *parents may lack knowledge of or have limited understanding* of the foci of educational systems, which in turn may limit their involvement (Alieva, 2021).

1. There is a very good synthesis of literature but try and be geographically specific. Where are the studies you have mentioned located? Are they still relevant to Ghana. ( I am sure they are but the specificity will help credence and authority.)

**Response**: Thank you for pointing this out. We have indicated where individual studies have been conducted.

1. “Although these patterns offer a diverse picture of caregiver involvement, previous studies have been limited to quantitative research methods that evaluate the association be” what patterns?

**Response**: We have expanded the sentence to indicate its focus on child characteristics that shape caregiver involvement (new text is italicized). Although *the evidence on how child characteristics shape caregiver engagement offers a diverse picture of their involvement*, most previous studies have been limited to quantitative research methods that evaluate the association between caregiver engagement and child outcomes.  

 5. “gross enrolment rate increasing from approximately 60% in 2010 to 103% in 2020” I think I know what the author means but worth clarifying how it is 103%

**Response**: We have included a footnote that clarifies why the gross enrolment is rate is above 100%. The gross enrolment rate as defined by the World Bank is the total number of students enrolled in the primary school regardless of age. Therefore, the gross enrolment rate includes students who are younger or older than the official age range for primary school and can exceed 100%. Rates above 100% means that total enrolment at the primary school exceeds the size of the official primary school-age population often due to grade repetition or late/early school entry.

 6. “However, learning outcomes remain low. For example, according to the 2020 Ghana Education Facts, only 21% of learners, with almost equal proportions of males (20%) and females (22%), demonstrated the expected foundational reading skills. Similarly, only 16% of learners, with an almost equal share of males (17%) and females (14%), demonstrated the expected foundational numeracy skills. – what age are these learning outcomes applied to? **Response**: Thank you for pointing this out. We have added the age range (new text is italicized. For example, according to the 2020 Ghana Education Facts, *among learners aged 7 to 14 years*, only 21%, with almost equal proportions of males (20%) and females (22%), demonstrated the expected foundational reading skills. Similarly, only 16% of learners, with an almost equal share of males (17%) and females (14%), demonstrated the expected foundational numeracy skills

 7. “although caregiver education has long been a key predictor of children’s learning outcomes, the narration of caregivers in our study suggests why it matters in this context.” Although the biggest impact factor is arguably income rather than education. Systemic factors are key.

**Response**: Thanks for your suggestion. We have not measured income in this setting given the challenges of accurately measuring incomes in high-informal economies. We focused on education as our main proxy for socioeconomic status to explore the links between “systemic issues” (which we interpret as access to economic, social, and informational resources) and engagement. We have clarified this point about socioeconomic status in the text (new text is italicized) Although caregiver education has long been a key predictor of children’s learning outcomes, the narration of caregivers in our study suggests why it matters in this context, *as it serves as a proxy for access to economic, social and informational resources available to the caregivers and their families*

# Reviewer report: Reviewer response for version 1

**DOI:** 10.21956/openreseurope.22194.r60755

**Published:** 2025-10-07

**Authors:** Janet Goodall

**Affiliations:** 1 Swansea University Library, Swansea, Wales, UK

Thank you for this paper - I enjoyed reading it and it will make a valuable contribution to the field. 

 This paper details qualitative work examining care givers' views on their engagement with young people's (5 - 15) schooling in three areas in Ghana.

 I have answered  'partly' to two sections - 

 1. In reference to the current literature - please see the comment below about qualitative studies.  In general, references to the literature are otherwise appropriate. 

 2.Conclusions - again, please see below.  There are two areas where comments in the discussion section do not appear in the data - first in reference to girls' household duties and secondly in relation to the quality of teaching.

 There are some more specific comments which I hope will strengthen the eventual indexing of this paper:

 Introduction:

 The sentence, “Caregivers’ inability to understand these changes may hinder their involvement, which can be exacerbated for those with little or no education” holds a deficit view which might be modulated – that is, are caregivers unable to understand these changes or are they not supported to do so?  Rewording this would support the otherwise asset based approach taken to caregivers in this paper.

 It would be useful early on in the paper to articulate how ‘education’ is defined, as it seems to relate only to school based learning.  That’s acceptable but this is clearly not the only purposeful learning undertaken by young people in the study, (a generally accepted definition of education) so clarification would be useful – the study is about how care givers interact with young people’s school based education/learning.

 In the section The Ghanian Context – how can school enrollment be above 100%? 

 While I agree that there is a need to investigate care givers’  perspectives, the link between these perspectives and outcomes (in the same section) could be strengthened for the reader.

 The idea that there is no research relating to parental engagement and child sex/age is not quite accurate  - see Alter, E., Fényes, H., & Pusztai, G. (2025). The multi‐faceted effects of gender in parental involvement: The results of a systematic literature review. *Review of Education*, *13*(2), e70091; Lee, S. M., Kushner, J., & Cho, S. H. (2007). Effects of parent's gender, child's gender, and parental involvement on the academic achievement of adolescents in single parent families. *Sex Roles: A Journal of Research, 56*(3-4), 149–157 and Muller, C. (1998). Gender differences in parental involvement and adolescents' mathematics achievement. *Sociology of Education*, 336-356, among others.

 It would be useful to add something to this section which gives a brief overview of the Ghanaian educational system  - c.f. ages for primary, junior secondary phases; the requirements or not of school fees, etc.

 The data collection and data analysis sections are thorough and clear.

 Findings:

 The paper does well to take an asset based approach to caregivers’ emphasis on providing basic needs – this can at times be dismissed by researchers but here it is shown in a positive, asset based light.

 It is interesting that the reports of caregivers’ comments often reference ‘duty’ – it would be interesting to unpack this choice of term.

 It is also interesting that the quotes chosen about caregivers attending school mention visiting the children at school to check that they are there, but do not mention much about interaction with teaching staff – is this representative of the findings?  This is an interesting difference to much of what is in the field already.

 Discussion:

 The paper might go further in relation to the discussion of male caregivers’ advice – do men in this culture give more advice because of the noted patriarchal nature of the culture – e.g. is men’s advice seen as more authoritative or impactful?  Is it given more credence by children and young people, perhaps?

 The discussion section mentions the higher opportunity cost of educating girls due to their involvement in household activities – but this does not appear reported in the data – a quotation to this effect might be added to the findings section?

 The suggestion that the quality of teaching may impact on children’s motivation does not appear to come from the qualitative data – so while the point may well be valid, its inclusion in this paper needs further justification.

 Final comment:

 Overall, this is a well written and clear paper which will add to the field.

If applicable, is the statistical analysis and its interpretation appropriate?

Not applicable

Is the study design appropriate and is the work technically sound?

Yes

Is the work clearly and accurately presented and does it engage with the current literature?

Partly

Are the conclusions drawn adequately supported by the results?

Partly

Are sufficient details of methods and analysis provided to allow replication by others?

Yes

Are all the source data and materials underlying the results available?

No source data required

Reviewer Expertise:

Parent, family and community engagement in learning

I confirm that I have read this submission and believe that I have an appropriate level of expertise to confirm that it is of an acceptable scientific standard.

# Response

**Published:** 2026-02-25

**Authors:** Esinam Avornyo

**Affiliations:** Institute of Education, University of Cape Coast College of Education Studies, Cape Coast, Central, Ghana

Introduction:

1. The sentence, “Caregivers’ inability to understand these changes may hinder their involvement, which can be exacerbated for those with little or no education” holds a deficit view which might be modulated – that is, are caregivers unable to understand these changes or are they not supported to do so? Rewording this would support the otherwise asset based approach taken to caregivers in this paper.

**Response**: We have re-written this statement, “ *Caregivers’ lack of knowledge of these changes or lack of school systems support to facilitate their understanding of these changes may hinder their involvement, and this can be exacerbated for those with little or no education. *

1. It would be useful early on in the paper to articulate how ‘education’ is defined, as it seems to relate only to school based learning. That’s acceptable but this is clearly not the only purposeful learning undertaken by young people in the study, (a generally accepted definition of education) so clarification would be useful – the study is about how care givers interact with young people’s school based education/learning.

**Response**: Thank you for your suggestion regarding the definition of ‘education’. We have specified the type of education in our introduction (new text is italicized). In particular, their involvement in children’s *formal, school-based education*, including school- and home-based practices, is a major goal of educational systems worldwide (Goodall, 2017; Smith, 2021).

1. In the section The Ghanaian Context – how can school enrollment be above 100%?

**Response**: Thank you for pointing this out. We have included a footnote that clarifies why gross enrolment is above 100%. The gross enrolment rate as defined by the World Bank is the total number of students enrolled in the primary school regardless of age. Therefore, the gross enrolment rate includes students who are younger or older than the official age range for primary school and can exceed 100%. Rates above 100% means that total enrolment at the primary school exceeds the size of the official primary school-age population often due to grade repetition or late/early school entry.

1. While I agree that there is a need to investigate care givers’ perspectives, the link between these perspectives and outcomes (in the same section) could be strengthened for the reader.

**Response**: We have included a sentence on the importance of the work in the section, The Ghanaian context (new text is italicized). Although the Ghana government is taking steps to improve caregiver involvement through initiatives such as the Ghana Accountability for Learning Outcomes (GALOP) framework (Ministry of Education, n.d.), for example, much less is known about how caregivers perceive their involvement in their children’s *formal education. Thus, evidence from this qualitative study contributes an in-depth understanding of the dynamics of caregiver involvement so that policies and programs can strengthen this important component of educational systems *

1. The idea that there is no research relating to parental engagement and child sex/age is not quite accurate - see Alter, E., Fényes, H., & Pusztai, G. (2025). The multi‐faceted effects of gender in *parental involvement: The results of a systematic literature review. Review of Education, 13(2), *e70091; Lee, S. M., Kushner, J., & Cho, S. H. (2007). Effects of parent's gender, child's gender, and parental involvement on the academic achievement of adolescents in single parent families. Sex Roles: A Journal of Research, 56(3-4), 149–157 and Muller, C. (1998). Gender differences in parental involvement and adolescents' mathematics achievement. Sociology of Education, 336-356, among others.

**Response**: Thank you for pointing this out and suggesting relevant literature. We have incorporated findings from the systematic evidence conducted by Alter et al. (2025).

1. It would be useful to add something to this section which gives a brief overview of the Ghanaian educational system - c.f. ages for primary, junior secondary phases; the requirements or not of school fees, etc.

**Response**: We have added that to our section on the Ghanaian context (new text is italicized. *Ghana is a lower-middle income country in West Africa. In Ghana, pre-tertiary education (also known as basic education) comprises a 2-6-3-3 system, which represents two years of kindergarten education for 4–5-year-olds, six years of primary education covering ages 6 to 11, three years of lower secondary education for 12–14-year-olds and three years of senior secondary education for ages 15 to 17.  At each level, free education is provided for children enrolled in public or government-owned schools.*

1. The data collection and data analysis sections are thorough and clear.

**Response**: Thank you Findings:

1. The paper does well to take an asset based approach to caregivers’ emphasis on providing basic needs – this can at times be dismissed by researchers but here it is shown in a positive, asset based light.

**Response**: Thank you for your positive comment.

1. It is interesting that the reports of caregivers’ comments often reference ‘duty’ – it would be interesting to unpack this choice of term.

**Response**: Thank you pointing this out. We have explained what the term ‘duty’ highlights in this context (new text is italicized) Most caregivers indicated that their key role in supporting their children’s education was to provide for their children’s basic needs. Essentially, *they viewed this as a “duty”, which reflects caregivers’ perception of their role not just as tasks, but a deep sense of responsibility and commitment* to ensuring that children were well-fed, clean, enrolled in school, and had basic school supplies such as a tidy uniform, books, and stationery.    

1. It is also interesting that the quotes chosen about caregivers attending school mention visiting the children at school to check that they are there, but do not mention much about interaction with teaching staff – is this representative of the findings? This is an interesting difference to much of what is in the field already.

**Response**: Thank you for pointing this out. In our narrative, we mentioned that caregivers’ school visits also allow them to “discuss their children’s progress with their teachers.” In our chosen quotes, participants talked about “finding out how their children are doing academically” and “know about their studies”. While the quotes do not explicitly mention engaging teachers, this will typically involve interacting with the teaching staff to know about the child’s progress. However, we have added a third quote to exemplify caregiver-teacher interactions. *Some of the children are very stubborn and for that reason some of us have created the time to visit the children at their various schools to be sure they are in school and learning very well. We ask the teachers how the children are performing in school to be able to advise them appropriately when they return from school. (Male R8, Eastern).* Discussion:

1. The paper might go further in relation to the discussion of male caregivers’ advice – do men in this culture give more advice because of the noted patriarchal nature of the culture – e.g. is men’s advice seen as more authoritative or impactful? Is it given more credence by children and young people, perhaps?

**Response**: Thank you. We have extended our discussion to reflect that. *In Ghana, male caregivers’ advice is important due to the country’s patriarchal social structure, where men traditionally hold significant authority as household heads and decision makers in critical matters like education.*

1. The discussion section mentions the higher opportunity cost of educating girls due to their involvement in household activities – but this does not appear reported in the data – a quotation to this effect might be added to the findings section?

**Response**: Thank you for noting this. We have added to the finding. *The quantity of household chores or work often allocated to a child was identified as barriers to caregiver involvement. Participants expressed their awareness of the potential negative effects of excessive household chores or work on children’s education, as this leaves them with limited time to study. Children were often expected to fetch water and sweep the house. In some instances, children also help their caregivers to sell late at night (common in the urban contexts of Accra) or required to run economic-related errands for their families. This potentially deprives the children of the necessary hours needed for study or complete their homework:* *When you give them a lot of work to do, it becomes difficult for them to learn or do their homework. If the work is measurable, they can finish and do their homework, but if it is otherwise, it will prevent them from doing their homework if it moves into the night because we don’t have enough touch lights. (Female R3, Middle)* *Consistent with previous evidence on the gendered distribution of child work in LMICs (Pells, 2016), more household chores seem to be disproportionately assigned to girls as compared with boys. Chores are seen as a key part of a girl’s broader “education”, as highlighted in the following transcript:* *You can’t say that because she is going to school, learning, so she won’t do the chores. If you do that, the child would become worse. If it is a boy, it would be done for him. This is not difficult. If he is the youngest at home let him throw the rubbish away and fetch water, that is a boy. For a girl, let her do the house chores. When she goes to school, let her concentrate at school, the chores at home she must perform as a girl. So that it won’t worry her in the future (Female R, Accra).*

1. The suggestion that the quality of teaching may impact on children’s motivation does not appear to come from the qualitative data – so while the point may well be valid, its inclusion in this paper needs further justification.

**Response**: Thank you for pointing this out. We have added a finding on that. *Additionally, caregivers felt the use of technological devices in schools is negatively impacting the quality of teaching in schools. One caregiver’s response summarized the participant’s concern, highlighting the need for government to restrict access to phones in schools:* *In the time past when there were not phones, they were teaching. When the smart phones were not in existence we were taught very well, when there was no computer, we were taught well. Now that we have computers and phones the teaching standards have been lowered. If the government can ban smart phones or whatever so that we can move forward, so that they can teach the children well. (Female R8, Accra).*
